# An RBD virus-like particle vaccine for SARS-CoV-2 induces cross-variant antibody responses in mice and macaques

**DOI:** 10.1038/s41392-023-01425-4

**Published:** 2023-04-29

**Authors:** Yuanyuan Li, Yanan Zhang, Yu Zhou, Yan Li, Jiao Xu, Yuanbao Ai, Lei Xu, Xiuli Xiao, Bo Zhang, Jing Jin

**Affiliations:** 1 Patronus Biotech Co. Ltd., Yantai, China; 2 Key Laboratory of Special Pathogens and Biosafety, Wuhan Institute of Virology, Center for Biosafety Mega-Science, Chinese Academy of Sciences, Wuhan, 430071 China

**Keywords:** Vaccines, Vaccines, Vaccines

**Dear Editor,**

Since early 2020, severe acute respiratory syndrome coronavirus 2 (SARS-CoV-2) has spread globally infecting over 500 million people and causing over 6 million deaths. The massive worldwide immunisation programmes and the arising clinical trial data present a unique opportunity to compare the vaccines and various delivery platforms (i.e. viral vector platform, mRNA platform and virus-like particle platform). As reviewed from the published clinical data,^1^ it is evident that the mRNA vaccines (mRNA-1273 from Moderna, BNT162b2 from Pfizer) and subunit protein/adjuvant vaccine (NVAX-CoV2373 from Novavax) were able to induce an average 2–4 fold greater neutralizing antibody (nAb) titer as compared to the human convalescent serum (HCS) control. On the other hand, the viral vectored vaccines (Ad26.COV2.S from Johnson & Johnson, ChAdOx1 nCoV-19 from AstraZeneca) and the inactivated vaccine (CoronaVac from Sinovac) induced less nAb than the HCS. Interestingly, CoVLP from Medicago, which uses a plant-based virus-like particle (VLP) platform technology, was able to induce a >10-fold increase in the nAb response as compared to HCS;^2^ suggesting VLP vaccine or arrayed antigen delivery is highly immunogenic.

We designed a vaccine, named RBDM, targeting the receptor-binding domain (RBD) of the SARS-CoV-2 Spike glycoprotein (S-protein), whereby the wild-type (WT; Wuhan strain) RBD is delivered in an array on a VLP (an RBD-VLP vaccine) produced using a split protein Tag/Catcher technology as described previously.^3^ The RBD protein and VLP carrier, depicted in Fig. 1a, were expressed at high titres in CHO cells (>4 g/L) and *E. coli* BL21 (DE3) cells (>3 g/L) respectively. Using a good manufacturing practice (GMP) compatible purification process, RBDM achieved a purity of >95% and endotoxin level of <5EU/mL (Fig. 1b). Furthermore, RBDM robustly formed particles as confirmed by dynamic light scattering (DLS) reading at 35.86 nm; high-performance liquid chromatography size exclusion chromatography (HPLC-SEC) analysis at 26.4 min retention time; as well as transmission electron microscopy (TEM) analysis (Fig. 1c, d, e). The affinity between RBD antigen and human Angiotensin-converting enzyme 2 (hACE2) was measured in vitro using a Biolayer Interferometry (BLI) assay. RBDM demonstrated the strongest binding affinity constant to hACE2 among all other designs, likely due to the avidity of the interaction when arraying antigen on a VLP (Supplementary Fig. S1). The S protein trimer (S-trimer), depicted in Fig. 1a, was selected as a “reference vaccine” since most first-generation SARS-CoV-2 subunit vaccines were designed based on S-protein antigen.

**Fig. 1:**
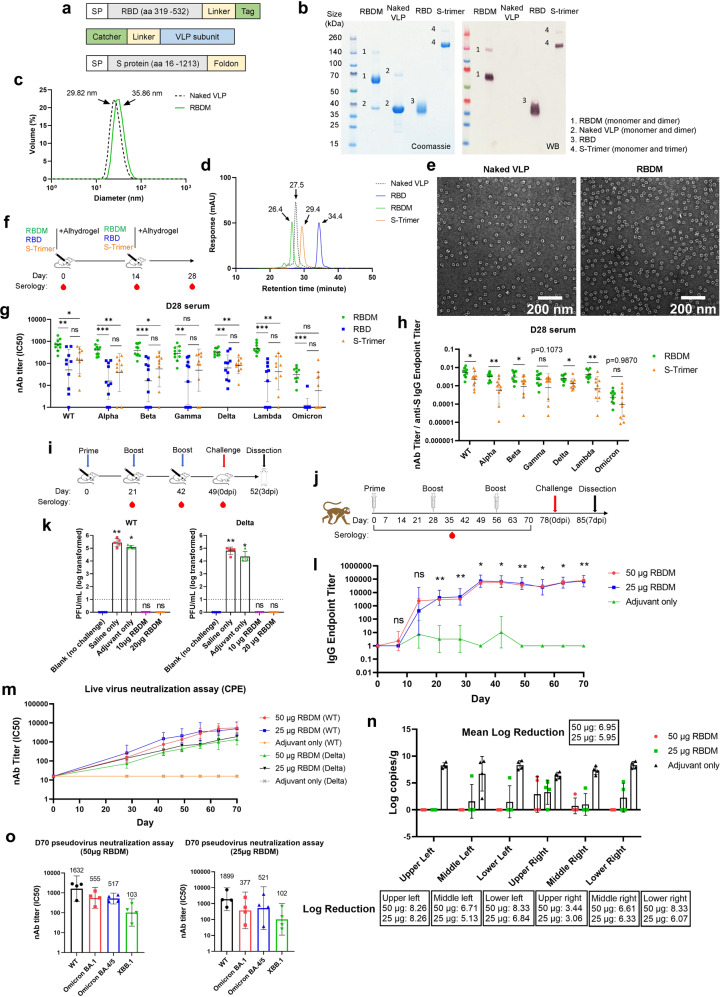
Development and characterization of an RBD virus-like particle vaccine against SARS-CoV-2. **a** Schematic representation of the RBD, VLP subunit and S-trimer design. SP signal peptide. **b** SDS-PAGE with Coomassie staining and anti-RBD western immunoblotting, reduced condition. **c** Dynamic light scattering (DLS), the average measurement of diameter is shown (*n* = 3 individual measurements). **d** High-performance liquid chromatography size exclusion chromatography (HPLC-SEC) analysis, retention time is shown. **e** Transmission electron microscopy (TEM) analysis, scale bar is 200 nm, magnification is ×9300. **f** The immune program of RBDM in mice. BALB/c mice (*n* = 10 per group) were immunised intramuscularly with either 5 µg of RBDM, RBD or S-Trimer, all formulated in Alhydrogel. Mice received two immunisations (day 0 and day 14), serum samples were collected on days 0, 14 and 28. Neutralizing antibody titer (nAb) was assessed in day 28 sera by ACE2 competition ELISA (**g**) and the ratio of nAb titer to IgG endpoint titer was calculated (**h**). **i** The immune and challenge program of RBDM in mice. K18-hACE2 transgenic mice (*n* = 10 per group) were immunised intramuscularly with either saline, adjuvant control, 10 μg or 20 μg of RBDM in Alhydrogel. Mice received three immunisations (day 0, day 21 and day 42), serum samples were collected on days 21, day 42 before immunisation and day 49 before challenge. Mice were challenged on day 49 and sacrificed 3 days post-infection (dpi) on day 52 for viral titer measurement in lung tissue. A naïve control group of mice that did not receive vaccination or challenge are called “blank”. The viral titer in the lung tissue 3 dpi was measured and is reported as plaque-forming units (PFU) per mL (**k**). **j** The immune and challenge program of RBDM in NHP. Rhesus macaques (*n* = 4 per group) were immunised intramuscularly with either adjuvant control, 25 μg or 50 μg of RBDM formulated in Alhydrogel. Each animal received three immunisations (day 0, day 28 and day 56), serum samples were collected throughout the study at the indicated time-points. Animals were challenged on day 78 and sacrificed on day 85 (7 dpi). Total IgG titers against Spike glycoprotein were measured (**l**), The neutralization antibody titer was measured by live virus neutralization assay (**m**) against WT and Delta variant. **n** Lung tissue samples were processed and the log reduction of sgRNA was calculated by subtracting the mean value of vaccinated group from the adjuvant group. **o** Anti-Omicron subvariant nAbs were measured on day 70 NHP serum by pseudovirus neutralization assay, the GMT values are labelled

We immunised BALB/c mice with two doses of RBDM formulated in Alhydrogel to evaluate its immunogenicity (Fig. 1f). RBDM was able to induce a significantly higher IgG response as compared to the soluble RBD; although it was not significantly different as compared to the larger S-trimer immunogen at each timepoint (Supplementary Fig. S2a). RBD-specific IgG subclasses were also measured in day 28 samples. Here, RBDM induced a significantly higher and more balanced IgG1, IgG2a and IgG2b response than the soluble RBD. A similar trend was observed when compared to S-trimer (Supplementary Fig. S2b). Neutralizing antibody (nAb) was measured using an ACE2-RBD binding competition ELISA. RBDM induced significantly higher nAb responses against most variants (Fig. 1g). For RBDM, the reduction of nAb titers from WT to variants were no more than 3-fold, expect for Omicron BA.1 where a >20-fold reduction was observed (Fig. 1g). To understand if the higher nAb response of the RBDM group was solely due to a higher total IgG response as demonstrated in the endpoint titre ELISA (Supplementary Fig S2a), we calculated the ratio of ELISA-measured nAb to IgG endpoint titer for individual animals. Per unit of endpoint titer, there were significantly more anti-WT, Alpha, Beta, Delta and Lambda nAbs in the RBDM vaccinated group than the S-trimer group. The nAb response against Gamma and Omicron BA.1 variant followed the same trend but was not statistically different (Fig. 1h). These data indicate the RBDM vaccine induced a qualitatively superior antibody response against most variants in our study.

To test if RBDM is effective against viral challenges, K18-hACE2 transgenic mice were immunised three times with 10 μg or 20 μg RBDM formulated in Alhydrogel adjuvant on days 0, 21 and 42 as depicted in Fig. 1i. Serum from individual mice was taken on days 21, 42 and 49. The IgG titers were measured and the vaccines at both doses induced significantly higher IgG response as compared to the saline and adjuvant control groups; while no significant difference was observed between the two vaccine doses (Supplementary Fig. S3a). Neutralizing antibody (nAb) was measured by pseudovirus assay and the nAb response against both the WT and Delta stains of pseudovirus were detected (Supplementary Fig. S3b, c). The mice were then challenged with the WT or Delta variant of SARS-CoV-2 live virus on day 49 followed by dissection on day 52. The viral loads in the lung tissue were measured by plaque assay and no live virus was detected in any of the vaccinated groups challenged with either strain of SARS-CoV-2 (Fig. 1k).

To test if RBDM is immunogenic in non-human primates (NHP), rhesus macaques were immunised three times with 50 or 25 μg RBDM or adjuvant control on days 0, 28 and 56; the immunisation strategy is depicted in Fig. 1j. The anti-Spike glycoprotein IgG was measured by ELISA and both dosage groups induced high antibody titers with similar trend, and both peaked after the second immunisation (Fig. 1l). Pseudovirus neutralization assay (Supplementary Fig. S4a) and live virus neutralization assay (Fig. 1m) were performed. Both tests confirmed induction of high nAb titers against the WT and Delta variant of SARS-CoV-2. The live virus neutralization assay against WT SARS-CoV-2 revealed relatively high nAb responses, with a geometric mean titer (GMT) of 3547 on day 56 after two vaccinations and 5552 on day 70 after three doses of RBDM. Comparing the preclinical studies of other vaccines utilising rhesus macaques as the NHP model, these results are higher than those reported, including Moderna’s mRNA-1273 with a highest GMT nAb of 3481;^4^ Pfizer’s BNT162b2 of 1689;^5^ AstraZeneca’s AZD1222 with a highest median titer of 74;^6^ a recombinant vaccine ZF2001 with GMT of 256;^7^ or Sinovac’s inactivated vaccine with a GMT of 50.^8^ The vaccine also induced a detectable IFN-γ T cell response, and a stronger IL-2 response, as revealed by ELISPOT assay on PBMC taken at the day 28 and 70 time-points (Supplementary Fig. S4b). Indeed, the strong immune response observed here conferred protection from heterologous Delta variant intranasal challenge. Rhesus macaques were challenged with the Delta variant of SARS-CoV-2 on day 78. We monitored the course of infection for 7 days by measuring the subgenomic RNA (sgRNA) in nasal swab, throat swab and anal swab. Vaccine at both doses reduced the level of detectable sgRNA (Supplementary Fig. S5a). The lung tissues were also extracted and analysed for viral sgRNA response; here we observed a substantial reduction of sgRNA in both the high and low dose vaccine groups as compared to the control group. A mean of 6.98 and 5.95 log reduction was achieved by the high dose and low dose vaccines, respectively (Fig. 1n). We also assessed lung tissues and scored each sample based on histopathological findings. Animals that received the control vaccine showed evidence of severe interstitial inflammation and pneumocyte hyperplasia in the lung, achieving a cumulative mean pathology score of 10.42 as compared to immunised animals with very mild and focal histopathological changes in a few lobes of lung (mean, 5.5 and 6.04) (Supplementary Fig. S5b and Supplementary Fig. S6). Following the global surge of the Omicron variants of SARS-CoV-2, day 70 sera were re-tested in a pseudovirus neutralization assay to analyze the fold difference of the nAb titers against WT to Omicron BA.1, Omicron BA.4/5 or XBB.1; we observed a reduction of nAb titer between 3- and 5-folds for Omicron BA.1 or BA.4/5, and an increased reduction of 16- to 19-folds for XBB.1 variant (Fig. 1o). Although the nAb titer against XBB.1 was significantly reduced, it is still encouraging to see a positive response considering the vaccine was designed from WT RBD. As the rhesus macaque model is closer to humans, such data give a strong indication the vaccine could work in human and would be effective against new viral variants.

In summary, RBDM described in this study is one of the few existing SARS-CoV-2 vaccine candidates that utilise VLP platform technology. It offers high immunogenicity and could afford protection against different variants in murine and NHP challenge models. Currently this vaccine is in multiple clinical trials, including a heterologous boost study, majority (>99%) of vaccine related adverse events observed so far were Grade 1–2, demonstrating this vaccine formulation is safe in human. We also anticipate the vaccine to be effective and one that should offer cross-protection against newly emerged variants.

## Supplementary information

Supplementary Materials

## Data Availability

The data used in the current study are available from the corresponding authors upon reasonable request.
