# Supplementary material for: An RBD virus-like particle vaccine for SARS-CoV-2 induces cross-variant antibody responses in mice and macaques
Source: Signal Transduct Target Ther. 2023 Apr 29;8:173. doi: 10.1038/s41392-023-01425-4 (PMC10147897; doi:10.1038/s41392-023-01425-4)
Supplement: Supplementary file 1 — Supplementary Materials [file 41392_2023_1425_MOESM1_ESM.docx]

Supplementary Materials for

An RBD virus-like particle vaccine for SARS-CoV-2 induces cross-variant antibody responses in mice and macaques

Yuanyuan Li, Yanan Zhang, Yu Zhou, Yan Li, Jiao Xu, Yuanbao Ai, Lei Xu, Xiuli Xiao, Bo Zhang and Jing Jin

Correspondence to: [liyuanyuan@luye.com](mailto:liyuanyuan@luye.com) ; [jinjing@luye.com](mailto:jinjing@luye.com)

**This PDF file includes:**

Materials and Methods

Figures. S1 to S6

**Material and Methods**

### Expression and purification of recombinant RBD-Tag and Catcher-VLP

The RBD (amino acids (aa) 319-532) of the WT (Wuhan) SARS-CoV-2 spike glycoprotein (protein sequence ID: QIA20044.1) was genetically fused to the N-terminus of the split-protein Tag ^1^. A mammalian signal peptide (SP) was added to the N-terminus of the construct. This RBD-Tag construct was then codon optimized and expressed in a Chinese hamster ovary (CHO) stable cell line as a secreted protein. After screening single cells for productivity, the highest producer was cultivated in shake flasks using a 13-day fed-batch process. From the clarified supernatant, high purity RBD-Tag was produced using a combination of orthogonal chromatographic methods on AKTA systems (Cytiva) and buffer exchanged using a tangential flow filtration (TFF) system equipped with Pellicon 2 Biomax 10 kDa MWCO membrane (Merck-Millipore). The C-terminus of the split-protein Catcher ^1^, was genetically fused to a flexible linker followed by the subunit of the VLP carrier as previously described ^2^. This Catcher-VLP (naked VLP) was codon optimised and expressed in *Escherichia coli* BL21 (DE3). Biomass was bulked up in an XDR-50 MO single-use fermenter (Cytiva) and harvested in Sorvall Lynx 6000 centrifuges (Thermo Fisher Scientific). Intracellular content was first released using an AH-PILOT high pressure homogenizer (ATS, China) and then clarified by a series of centrifugations. Naked VLP with high purity was produced using a combination of orthogonal chromatographic methods on AKTA systems (Cytiva) and buffer exchanged using a TFF system equipped with Pellicon 2 Biomax 300 kDa MWCO membrane (Merck-Millipore). To conjugate, the naked VLP and RBD were mixed at a fixed molar ratio and uncoupled RBD was removed from the conjugated VLP by size-exclusion chromatography (SEC) using a HiLoad 16/600 Superdex 200 pg column (Cytiva) on an AKTA system (Cytiva). After separation, the RBD-VLP (RBDM) was analyzed on sodium dodecyl sulfate polyacrylamide gel electrophoresis (SDS-PAGE) to determine the coupling efficiency by densitometry as previously described ^3^.

For the S-trimer, a foldon sequence (YIPEAPRDGQAYVRKDGEWVLLSTFL) was added to the C terminus of WT SARS-CoV-2 spike glycoprotein (protein sequence ID: QIA20044.1) (aa 15-1213) followed by a polyhistidine tag. Multiple mutations were applied to generate this prefusion-stabilized trimeric structure as described previously ^4^.

### SDS-PAGE and Western Blotting

For SDS-PAGE, NuPAGE 4-12% Bis-Tris Midi Gel (Thermo Fisher Scientific) was used for all the analyses in this study. Before loading, protein samples were mixed with NuPAGE LDS Sample Buffer (Thermo Fisher Scientific) containing 10mM DTT and denatured at 70 °C for 5 min. Electrophoresis was performed at 150 V for 1 h in an XCell4 SureLock Midi-Cell (Thermo Fisher Scientific) filled with 1× NuPAGE MES SDS Running Buffer (Thermo Fisher Scientific). Gels were then stained with InstaBlue Protein Stain Solution (APExBIO) and imaged using Amersham ImageQuant 800 system (Cytiva).

For Western blot, after SDS-PAGE, gels were transferred onto a 0.2 µm nitrocellulose membrane using Trans-Blot Turbo Mini Transfer Pack (BIO-RAD) at 25 V for 7 min in a Trans-blot Turbo Transfer system (BIO-RAD). After transfer, the membrane was then blotted using an iBind Automated Western Systems (Thermo Fisher Scientific). Briefly, the blot was blocked in 1× iBind Flex working solution (Thermo Fisher Scientific) followed by incubation with primary anti-SARS-CoV-2 Spike RBD antibody at 1:2000 dilution (Arigo Biolaboratories) and secondary antibody of goat anti-mouse IgG alkaline phosphatase conjugate at 1:1000 dilution (Sigma-Aldrich). Colorimetric signal was developed by incubation with SIGMAFAST BCIP/NBT (Sigma-Aldrich) solution as a substrate.

### BCA Assay

Bicinchoninic acid (BCA) assay was performed using Pierce BCA Protein Assay Kit (Thermo Fisher Scientific) for total protein quantification according to the manufacturer’s instruction. Briefly, a series of bovine albumin standards were diluted to 0.025-1.5 mg/mL using the same diluent as the sample. 25 µL of each standard and sample were pipetted into a Nunc Microwell 96F plate (Thermo Fisher Scientific) in triplicates and incubated with working reagent at 37 °C for 30 min. Absorbance at 562 nm was measured using an Infinite 200 PRO plate reader (Tecan) and plotted to construct a standard curve for sample evaluation.

### Dynamic Light Scattering (DLS)

Dynamic light scattering (DLS) was performed using Malvern Zetasizer Lab (Malvern) equipped with a 633-nm He-Ne laser. Size measurements were operated at an angle of 90° and data were collected and analysed using ZS XPLORER (Malvern). Approximately 1 mL of the sample (at 0.25 mg/mL) was measured in a DTS0012 disposable polystyrene cuvette (Malvern) at a controlled temperature of 25 °C. Viscosity and refractive index at 25 °C were adjusted accordingly. Testing of each sample was repeated 3 times.

### HPLC

Size-exclusion high-performance liquid chromatography (HPLC) was performed on a 1260 Infinity II Bio-Inert LC system (Agilent Technologies) equipped with SRT SEC-1000 column (300.0 mm × 7.8 mm i.d.) and Zenix-C SEC-150 column in tandem (150.0 mm × 7.8 mm i.d.) (Sepax Technologies). Columns were first equilibrated with two column volumes (CV) of mobile phase, 0.2M Arginine in 50 mM Phosphate Buffer, pH 7.0. For each run, 50 µL sample was injected and isocratic elution was performed at a flow rate of 0.4 mL/min at room temperature (RT). UV signal detection at 280 nm was collected for analysis of peak retention.

### Transmission Electron Microscopy (TEM)

3-4 µL naked VLP or RBDM sample at a concentration of 100 ng/μL was adsorbed onto a glow-discharged carbon-coated copper grid for 1 min. The grid was then washed with Milli-Q water and blotted to dry. Negative staining was performed with 0.75% uranyl formate for 1 min. Electron micrographs of VLP or RBDM were recorded using an FEI Tecnai 12 transmission electron microscope (Thermo Fisher Scientific) equipped with an Orius SC200 CCD camera operating at 120 kV.

### Biolayer Interferometry (BLI)

A BLI analysis was performed on a GatorPrime instrument (Gator Bio) at 30 °C with shaking at 1000rpm. Signals were collected at the default frequency of 5.0 Hz.

### Immunisation

Six to eight weeks old female BALB/c mice purchased from Ruige biotech, housed in specific-pathogen free (SPF) environments, were vaccinated with 5 µg total dose of vaccine (split over both legs) via the intramuscular (i.m.) route, using a prime-boost-boost regime (prime on day 0, boost on day 14 and day 28). RBD, S-trimer and RBDM immunogens were all formulated in Alhydrogel adjuvant (Invivogen) at 7.5 μg per dose and incubated at RT for 1 h before injection. Blood samples were collected on days 14, 28, 35 and 42, and sera were obtained from whole blood by leaving samples overnight at 4 °C to clot, followed by 10 min centrifugation at 16,000 × g at RT. Sera were pipetted into fresh Eppendorf tubes and frozen until further analysis.

Five weeks old female K18-hACE2 transgenic mice ^5^ obtained from GemPharmatech were housed in a SPF environment. Mice received high dose (20 µg), low dose (10 µg) of RBDM and Alhydrogel control or saline control via the i.m. route on days 0, 21 and 49; blood samples were collected on days 21, 42, 49 and sera were processed similarly as for BALB/c mice. After vaccination, the mice were transferred to a biosafety level 3 environment for the viral challenge study.

Three to four years old male and female rhesus macaques were housed in a SPF environment and were immunised with high dose (50 µg), low dose (25 µg) of RBDM and Alhydrogel control via the i.m. route on days 0, 28 and 56. Blood samples were collected at multiple timepoints, as described in Results, to obtain sera for immunogenicity analysis. After vaccination, rhesus macaques were transferred to a biosafety level 3 environment for the viral challenge study.

### IgG Endpoint ELISA

Anti-SARS-CoV-2 total IgG endpoint titer of serum collected from immunised animals was determined by indirect ELISA assay. 96-well Nunc MaxiSorp plates (Thermo Fisher Scientific) were coated with SARS-CoV-2 Spike (S) glycoprotein trimer (AcroBiosystem) at 100 ng/50 μL/well for 1 h at RT. After washing two times with 0.05% Tween 20 in PBS (PBST), the plates were blocked with Blocker Casein in PBS (Thermo Fisher Scientific) at 250 μL/well for 1 h at RT before washing two times again with PBST. Serial diluted sera were applied to each well for 1 h at RT, followed by incubation with 1:5000 dilution of goat anti-mouse IgG conjugated with HRP (Abcam) or goat anti-monkey IgG conjugated with HRP (Abcam) for 1 h at RT. After a further six times washing with PBST, the plates were developed using Tetramethylbenzidine (TMB) (Thermo Fisher Scientific, USA) for 10 min at RT. Subsequently, 1 N HCl was added to stop the reaction, the absorbance was recorded, and the value was read as 450 nm - 620 nm. The endpoint titer is defined as the X-axis intercept of the dilution curve at an absorbance value (±three standard deviations or 0.15, whichever was higher) greater than the optical density (OD) for a naïve serum.

For the IgG subclass endpoint ELISA, HRP conjugated anti-mouse IgG1, IgG2a, IgG2b or IgG3 secondary antibodies were used (IgG1, IgG2a from Abcam, UK; IgG2b, IgG3 from Frdbio).

### ACE2-competition ELISA

96-well Nunc MaxiSorp plates (Thermo Fisher Scientific) were coated with 200 ng/50 μL/well ACE2-Fc (AcroBiosystem) at 4 °C overnight. After washing two times with PBST, the plates were blocked with Blocker Casein in PBS (Thermo Fisher Scientific) at 250 μL/well for 1 h at RT before washing two times with PBST.

50 ng/mL biotinylated SARS-CoV-2 Spike RBD protein of WT or different variants (AcroBiosystem) were mixed 1:1 (v/v) with streptavidin-HRP (Abcam) (1:10,000 diluted in blocking solution) for 1 h at RT. The mixture was added to the plate (50 µL per well) with serial diluted sera (50 µL per well) and then incubated for 1.5 h at RT. After washing six times with PBST, TMB (Thermo Fisher Scientific) was added for signal development for 20 min at RT, followed by 1 N HCl addition to stop the reaction. The absorbance was recorded, and the value was read as 450 nm - 620 nm.

The percentage of inhibition was calculated as follows: % inhibition = (1-P/A) × 100 %, where A is the OD signal of RBD binding to ACE2-Fc when no serum was present, and P is the OD signal of RBD binding to ACE2-Fc in presence of serum at a given dilution. The IC50, defined as the serum dilution that gives 50 % binding inhibition, of a given serum sample was calculated using non-linear regression, i.e. log (inhibitor) vs. response (four parameters), in Prism 9 (GraphPad Software).

### Pseudovirus neutralisation assay

Pseudovirus from GenScript and Vazyme were purchased to measure the neutralizing antibody response against viral variants. In brief, pseudovirus carrying a luciferase reporter and encapsulated in WT or variant spike proteins was incubated with serial dilutions of the serum sample for 1 h at 37 °C. The mixture was then added to a culture of HEK293/ACE2 cells or Vero cells in 96-well plates and incubated in a humidified cell culture chamber at 37 °C with 5 % CO_2_ for 24-48 h according to manufacturers’ protocols. Medium was removed at the end of the incubation, and 100 μL of luciferase detection reagent was added to each well. Luminescence in relative light units (RLU) was measured by an Infinity M Plex luminometer (Tecan). The dose-response curves were plotted with the relative luminescence unit against the sample concentration. Non-linear regression was performed with a 4-parameter model using Prism 9 (GraphPad Software), the IC50 was calculated as a 50 % reduction in RLU.

### Live virus neutralization assay

Live virus neutralisation assays were performed as described previously ^6^. To assess the neutralization of the SARS-CoV-2 WT and delta variant (B1.617.2) infection, Vero E6 cells (5 × 10^4^) were seeded in 96-well plates and grown overnight. SARS-CoV-2 at the 50 % tissue-culture infectious dose (TCID_50_) was preincubated with an equal volume of diluted sera from immunised mice or monkeys before addition to cells. After incubation at 37 °C for 1 h, the mixture was added to Vero E6 cells. On day 3 after infection, cytopathic effects were recorded under the microscope and the neutralizing titres of the dilutions of sera that resulted in complete or EC_50_ inhibition were calculated.

### ELISPOT assay

Rhesus macaque peripheral blood mononuclear cells (PBMC) were taken on dayd 0, 28 and 70, and the assay was performed using monkey IFN-γ, IL-2 and IL-6 ELISPOT kit (MabTech). In brief, the pretreated plates were washed with Dulbecco’s PBS (DPBS) and blocked with complete media for 30 min at RT. WT SARS-CoV-2 RBD peptides (aa 319-532, 20 aa in length with 10 aa overhangs, synthesis by GenScript) were prepared and plated at 1µg per peptide per well, a total of 21 peptides were used. A total of 200,000 PBMC were added to each well and incubated at 37 °C, 5 % CO_2_ for 24 h. The next day, plates were washed with DPBS, biotinylated mAb specific for IFN-γ, IL-2 and IL-6 (MabTech) were added in separate wells and incubated at RT for 2 h followed by wash and incubation with Streptavidin-ALP (MabTech) for an additional 1 h. A final wash step was followed by addition of development solution BCIP/NBT-plus for 10 min. Plates were washed with water to stop development and dried before reading by ELISPOT reader (AID).

### K18-hACE2 mice challenge study

WT SARS-CoV-2 (IVCAS 6.7512) and the Delta variant (IVCAS 6.7593) were isolated at Wuhan Institute of Virology from COVID-19 patients. The two viruses were all amplified and titrated by standard plaque forming assay on Vero-E6 cells. Vero-E6 cells were cultured in Dulbecco’s modified Eagle’s medium (DMEM) containing 10 % fetal bovine serum (FBS), 100 units/mL of penicillin and 100 μg/mL of streptomycin and maintained at 5% CO_2_ at 37 °C.

After vaccination, K18-hACE2 mice were anaesthetized with Avertin (250 mg/kg) and intranasally infected with 1×10^5^ plaque-forming units (PFU) WT or Delta SARS-CoV-2 strain in a total volume of 50 μL DMEM. Mice were weighed and observed for clinical signs daily throughout the study. At day 3 post-SARS-CoV-2 infection, mice were sacrificed and the lung tissues were collected for viral load determination and histological analysis. Lungs of mice were homogenized in DMEM, then clarified by centrifugation at 13, 000 rpm for 10 min. The supernatants of lung homogenates were assayed for viral load by plaque assay as described previously ^7^.

### Non-human primate challenge study

SARS-CoV-2 Delta variant was obtained from the Chinese CDC and amplified using Vero-E6 cells. After 72 h of culturing, when CPE effect was observed, the virus was centrifuged and clarified by TFF method followed by TCID_50_ assay to measure the viral titer.

After three immunisations, rhesus macaques were challenged with 1×10^6^ TCID_50_ SARS-CoV-2 Delta variant intranasally on day 78. At 1, 3, 5 and 7 days-post infection (dpi), nasal swab, throat swab and anal swab were performed. At 7 dpi rhesus macaques were sacrificed and the lungs were dissected and lung tissues were homogenised. The viral loads (sgRNA level) in the swabs and lung tissues were measured by RT-qPCR. The primer and probe sequences used were derived from the N gene, according to the sequences recommended by WHO and China CDC: forward 5’- GAC CCC AAA ATC AGG GAA AT -3’; reverse, 5’- TCT GGT TAC TGC CAG TTG AAT CTG -3’; probe, 5’- FAM – ACC CCG CAT TAC GTT TGG TGG ACC - BHQ1 - 3’). For paraffin-embedded sections, tissues were collected, fixed in 10 % neutral-buffered formalin, and embedded in paraffin, and 5μm sections were prepared for standard haematoxylin and eosin staining.

### Statistical Analysis

Depending on the number of groups involved, statistical analysis was performed using either a unpaired t test (to compare two groups of data) or Kruskal Wallis test followed by Dunn’s multiple comparison post-test (to compare three or more groups of data). The statistical tests were performed in Prism 9 (GraphPad Software) and *p* values less than 0.05 were considered significant.

### References

1 Fougeroux, C. *et al.* Capsid-like particles decorated with the SARS-CoV-2 receptor-binding domain elicit strong virus neutralization activity. *Nat Commun*. **12**, 324, (2021).

2 Hsia, Y. *et al.* Design of a hyperstable 60-subunit protein icosahedron. *Nature*. **535**, 136-139, (2016).

3 Bruun, T. U. J., Andersson, A. C., Draper, S. J. & Howarth, M. Engineering a Rugged Nanoscaffold To Enhance Plug-and-Display Vaccination. *ACS Nano*. **12**, 8855-8866, (2018).

4 Hsieh, C.-L. *et al.* Structure-based design of prefusion-stabilized SARS-CoV-2 spikes. *Science*. **369**, 1501-1505, (2020).

5 Yinda, C. K. *et al.* K18-hACE2 mice develop respiratory disease resembling severe COVID-19. *PLoS Pathog*. **17**, e1009195, (2021).

6 Yang, J. *et al.* A vaccine targeting the RBD of the S protein of SARS-CoV-2 induces protective immunity. *Nature*. **586**, 572-577, (2020).

7 Zhang, Y.-N. *et al.* A mouse model for SARS-CoV-2 infection by exogenous delivery of hACE2 using alphavirus replicon particles. *Cell Res*. **30**, 1046-1048, (2020).

**Figure S1. Antigenicity characterization of RBD monomer, S-Trimer and RBDM.**

Biolayer interferometry (BLI) kinetic assays of RBD monomer, S-Trimer and RBDM against ACE2 were performed. KD: binding affinity constant calculated as K_on_/K_off_; smaller values generally indicate a stronger binding ability. K_on_: association rate. K_off_: dissociation rate.

**Figure S2. Immunogenicity of RBDM in BALB/c mice.**

BALB/c mice (n=10 per group) were immunised intramuscularly with either 5 µg of RBDM, RBD or S-Trimer, all formulated in Alhydrogel. Mice received two immunisations (day 0 and day 14), serum samples were collected on days 0, 14 and 28. (**a**) S protein-specific total IgG titers were measured by endpoint ELISA for each timepoint and (**b**) S protein-specific IgG subclasses were also measured on day 28 samples. Each dot represents an individual sample; bars represent GMT ± 1 SD. Kruskal Wallis test followed by Dunn’s multiple comparisons test was performed; ns means not significant, * *p<0.05*, ** *p<0.01*, *** *p<0.001*, **** *p<0.0001*.

**Figure S3. Immunogenicity of RBDM in K18-hACE2 transgenic mice.**

K18-hACE2 transgenic mice (n=10 per group) were immunised intramuscularly with either saline, adjuvant control, 10 μg or 20 μg of RBDM in Alhydrogel. Mice received three immunisations (day 0, day 21 and day 42), serum samples were collected on days 21, day 42 before immunisation and day 49 before challenge. Mice were challenged on day 49 and sacrificed 3 days post-infection (dpi) on day 52 for viral titer measurement in lung tissue. A naïve control group of mice that did not receive vaccination or challenge are called “blank”. (**a**) Total IgG titers were measured by endpoint ELISA against Spike glycoprotein for each time-point. The neutralization antibody titer against WT strain (**b**) and Delta strain (**c**) were measured by pseudovirus neutralization assay with IC50 readout. Each dot represents an individual sample; bars represent GMT ± 1 SD. Kruskal Wallis test followed by Dunn’s multiple comparison post-test was performed; all groups were compared with the adjuvant only group; for E, all groups were compared with the blank group; ns means not significant, ** p<0.05*, *** p<0.01*, **** p<0.001*, ***** p<0.0001*.

**Figure S4. Immunogenicity of RBDM in rhesus macaques.**

Rhesus macaques (n=4 per group) were immunised intramuscularly with either adjuvant control, 25 μg or 50 μg of RBDM formulated in Alhydrogel. Each animal received three immunisations (day 0, day 28 and day 56), serum samples were collected throughout the study at the indicated time-points. Animals were challenged on day 78 and sacrificed on day 85 (7 dpi). (**a**) Total IgG titers against Spike glycoprotein were measured by endpoint ELISA for each time-point. The neutralization antibody titer was measured by pseudovirus neutralization assay. (**b**) Expression of IFN-γ and IL-2 from PBMC stimulated with RBD peptides. For **a**, GMT ± 1 SD is shown; for **b**, each dot represents an individual sample and the bar represents mean ± 1 SD. Kruskal Wallis test with Dunn’s multiple comparisons post test was performed for **b**; ns means not significant, * p<0.05.

**Figure S5. RBDM protects rhesus macaques from Delta strain virus challenge.**

Rhesus macaques were challenged with 1E6 TCID50 Delta variant of live SARS-CoV-2 virus on day 78 (0 dpi) and scarified on day 85 (7 dpi). (**a**) Levels of sgRNA after challenge from nasal swab, throat swab and anal swab was measured. (**b**) Lung tissue samples were processed and pathology scores for lung samples were measured. For **a**, mean ± 1 SD is shown; for **b**, each dot represents an individual sample and the bars represent the mean ± 1 SD.

**Figure S6. Representative histopathology of lungs from vaccinated NHP.**

Histopathological examinations in lungs were carried out from the Delta strain challenged macaques at 7 dpi. Lung tissue was collected and stained with hematoxylin and eosin. There was no evidence of exacerbated lung inflammation in RBDM immunized animals.
